# Deoxynivalenol Induces Apoptosis via FOXO3a-Signaling Pathway in Small-Intestinal Cells in Pig

**DOI:** 10.3390/toxics10090535

**Published:** 2022-09-13

**Authors:** Tae Hong Kang, Kyung Soo Kang, Sang In Lee

**Affiliations:** 1 Department of Animal Science and Biotechnology, Kyungpook National University, Sangju-si 37224, Korea; 2 Department of Bio Life Sciences, Shingu College, Seongnam-si 13174, Korea

**Keywords:** apoptosis, deoxynivalenol, differentially expressed genes, Forkhead box

## Abstract

Deoxynivalenol (DON) is a mycotoxin that is found in feed ingredients derived from grains such as corn and wheat. Consumption of DON-contaminated feed has been shown to cause damage to the intestine, kidneys, and liver. However, the molecular mechanism by which DON exerts its effect in the small intestine is not completely understood. As a result, we profiled gene expression in intestinal epithelial cells treated with DON and examined the molecular function in vitro. We hypothesized that DON could induce apoptosis via the FOXO3a-signaling pathway in intestinal epithelial cells based on these findings. DON induced the apoptosis and the translocation of *FOXO3a* into the nucleus. Moreover, the inhibiting of *FOXO3a* alleviated the apoptosis and expression of apoptosis-related genes (*TRAL*, *BCL-6*, *CASP8*, and *CASP3*). ERK1/2 inhibitor treatment suppressed the translocation of *FOXO3a* into the nucleus. Our discovery suggests that DON induces apoptosis in intestinal epithelial cells through the FOXO3a-signaling pathway.

## 1. Introduction

Deoxynivalenol (DON), a *Fusarium* mycotoxin, is a risk factor for human and farm animal health, and the consumption of cereal-based foods can result in its exposure [1,2]. It has a high detection rate worldwide and is frequently present in feedstuffs [3]. DON can also enter the respiratory tract during baking, grinding, or brewing, posing a health risk to workers [4]. Porcine livestock are particularly susceptible to DON exposure due to their cereal-rich diets and rapid absorption [5,6]. Additionally, DON exposure can induce the expression of cytokines, chemokines, and inflammatory genes in vitro, while oral exposure can result in gastroenteritis, diarrhea, vomiting, anorexia, malabsorption, and weight loss [7]. According to adverse studies, DON causes oxidative stress, DNA damage, cell cycle arrest in the G2/M cell cycle phase, deregulation of cell signaling, intestinal barrier disorder, and apoptosis [8,9].

The intestinal epithelial cells (IECs) consist of a continuous single-layered sheet that serves as a barrier between the internal and external environments [10]. IECs are primarily responsible for transporting nutrients, electrolytes, and digestive enzymes, as well as acting as a protective barrier against pathogens and toxins [11]. The porcine intestinal epithelial cells (IPEC-J2) are critical for studying the transport and barrier properties of the porcine intestine at the subcellular or molecular level, thereby assisting in the reduction in animal usage [12]. Additionally, IPEC-J2 studies have been conducted in various fields, including cell viability, cell cycle, nutrient-transport genes, and mitochondrial-related genes [13]. However, the study of the apoptosis-related signaling pathways in IPEC-J2 following DON treatment is lacking.

Apoptosis, one of the cell death processes, is a highly regulated process of cell death that is triggered by a variety of signaling cascades and that ultimately results in death. Essentially, apoptosis is a defense mechanism that eliminates unnecessary cells [14,15]. On the other hand, excessive apoptosis has been linked to a variety of diseases, including neurological disorders, cardiovascular disorders, autoimmune disease, Crohn’s disease, and chronic inflammation [16,17]. Similarly, excessive cell death in IECs can disrupt the internal integrity of the intestinal barrier, resulting in inflammatory disease [18]. One of the apoptosis markers, caspases, are a critical regulator of cell death, and their activated form, cleaved caspase, has been shown to induce apoptotic cell death [19]. Numerous studies indicate that DON induces apoptosis [20,21].

The *Forkhead box O* (*FOXO*) transcription factor is involved in autophagy, apoptosis, cell cycle, differentiation, DNA damage repair, and oxidative stress [22]. In comparison to the other *FOXO* family members, *FOXO3a* is the most important functional transcription factor in cell death, which is associated with apoptosis and cell cycle inhibition [23]. *FOXO3a* induces transcription of apoptotic-associated genes such as *TRAIL*, *BCL-6*, *PUMA*, and *BIM*, resulting in apoptotic processes [24]. The *FOXO3a* transcription factor is also referred to as a tumor suppressor, and it has been implicated in a variety of signaling pathways, including PI3K/AKT and ER-α [23]. However, the molecular mechanism by which DON induces apoptosis in the small intestine is unknown and requires further investigation.

In this study, we aimed to identify the molecular mechanism on deoxynivalenol-mediated injuries in the porcine small-intestinal epithelium. Accordingly, gene-expression profiling was performed, and based on this data, genes differentially expressed by DON were identified, and the damage mechanism was additionally investigated.

## 2. Materials and Methods

### 2.1. Cell Culture and Treatment

IPEC-J2 (DSMZ, Braunschweig, Germany), which was originally isolated from a piglet’s jejunal epithelium, was cultured in a CO_2_ incubator using Dulbecco’s Modified Eagle Medium (DMEM) (Thermo Fisher Scientific, Wilmington, DE, USA) supplemented with 10% fetal bovine serum and 1% penicillin–streptomycin.

### 2.2. Cell Viability

Cell viability was determined using a microplate reader set to 450 nm absorbance after 2 h of treatment with WST-1 (Roche Diagnostics GmbH, Mannheim, Germany). Following the IPEC-J2 culture, cells were seeded at a density of 1 × 10^4^ per 100 μL in 96-well plates for 32 h and then incubated overnight in DMEM. For 15 h, DON (Sigma–Aldrich, St. Louis, MO, USA) treatments were performed at concentrations of 0.25 μg/mL, 0.5 μg/mL, 1 μg/mL, 2 μg/mL, 4 μg/mL, 10 μg/mL, 20 μg/mL, and 50 μg/mL.

### 2.3. Gene-Expression Profiling

IPEC-J2 cells were seeded at a density of 4 × 10^5^ in 6-well plates, incubated in DMEM overnight, and treated with DON 2 μg/mL. TRIzol reagent (Thermo Fisher Scientific, Wilmington, DE, USA) was used to extract total RNA. The Agilent 2100 bioanalyzer was used in conjunction with the RNA 6000 Nano Chip (Agilent Technologies, Amstelveen, The Netherlands) and the Thermo Inc. ND-2000 Spectrophotometer (Thermo, Wilmington, DE, USA) to determine the quality and quantity of RNA. The QuantSeq 3′ mRNA-Seq Library Prop kit was used to construct the library according to the manufacturer’s instructions for the controlling and testing of RNAs. To summarize, 500 ng total RNA was prepared and then hybridized to an oligo-dT primer containing an Illumina-compatible sequence at its 5′ end followed by reverse transcription. Following degradation of the RNA template, second-strand synthesis was initiated using a random primer with an Illumina-compatible linker sequence at its 5′ end. The double-strand library was purified using magnetic beads and amplified to incorporate all of the required adapter sequences for cluster generation. The completed library was purified from PCR components. Sequencing at a high-throughput single-end 75 sequencing was carried out using NextSeq 500 system (Illumina, Inc., San Diego, CA, USA). Gene-expression-profiling data were identified via Excel-based DEGs (differentially expressed genes) analysis. DEGs were analyzed using a GO (Gene Ontology) and KEGG (Kyoto Encyclopedia of Genes and Genomes) mapper for annotation, integration, and visualization. The DEGs were adjusted up- and downregulated by at least 2-fold.

### 2.4. Annexin-V and Propidium Iodide Staining

After 24 h of DON treatment, the IPEC-J2 cells were harvested and subsequently washed in PBS. Following centrifugation, the supernatants were discarded and treated with 1× Annexin binding buffer, and 5 μL Alexa Fluor 488 Annexin-V (Thermo Fisher Scientific, Wilmington, DE, USA) and 1 μL 100 mg/mL PI working solution were added. Following that, the cells were incubated at room temperature in the dark for 15 min. Subsequently, cells were stained with DAPI (Vector Laboratories, Burlingame, CA, USA) and mounted on slides using coverslips. The photographs were taken with a fluorescence microscope (Korealabtech, Seongnam-si, Gyeonggi-do, Korea).

### 2.5. Extraction of Cytoplasmic and Nuclear Proteins

Cytoplasmic and nuclear proteins were extracted according to the manufacturer’s instructions using NE-PER Nuclear and Cytoplasmic Extraction Reagents (Thermo, Wilmington, DE, USA). The cells were washed with chilled PBS and centrifuged for 3 min at 500× *g*. After discarding the supernatant, CERI (cytoplasmic extraction reagent) was added to the cell pellet and vortexed for 15 s. The suspension was then incubated on ice for 10 min. After adding the CERII in suspension and vortexing for 15 s, the mixture was incubated for 1 min. The suspension was centrifuged at 16,000× *g* for 5 min, and the cytoplasmic extraction suspension was transferred to a pre-chilled tube. The insoluble pellet was added with nuclear extraction reagent and vortexed for 15 s every 10 min for a total of 40 min. Following the ten-minute centrifugation at 16,000× *g*, the supernatant was transferred to a pre-chilled tube. The extracted protein was used in subsequent experiments.

### 2.6. RT-PCR and Western Blotting

The AccuPreP Universal RNA Extraction kit was used to extract total RNA (BioNEER, Daejeon, Korea). The cDNA was used to generate total RNA (1 μg) using the DiaStar™ RT Kit (SolGent, Daejeon, Korea). Primer 3 (http://frodo.wi.mit.edu) was used to design the primers for the target genes for qPCR. The following conditions were used for the qRT-PCR: 95 °C for 3 min, followed by 40 cycles at 95 °C for 15 s, 56–58 °C for 15 s, and 72 °C for 15 s. Normalized to glyceraldehyde-3-phosphate dehydrogenase (*GAPDH*), target gene levels were calculated using the 2^−ΔΔCt^ method. The primer sequence of genes were shown in Table 1.

Protein was extracted for Western blotting using a lysis buffer containing a protease inhibitor cocktail. A Pierce BCA Protein Assay kit (Thermo Fisher Scientific, Waltham, MA, USA) was used to determine the protein concentration. After electrophoresis in 9% polyacrylamide gel for 1 h at 100 volts, the protein sample was transferred to a membrane and washed three times with Tris-buffered saline containing 0.1% Tween-20. After 1 h of blocking, the membrane was processed overnight with the primary antibodies, including anti-FOXO3a (Novus Biologicals, Centennial, CO, USA) and anti-ERK1/2 (Cell Signaling Technology, Danvers, MA, USA). After 1 h of secondary antibody treatment, the membrane was visualized for immunoreactivity using the ECL reagent. The ChemiDoc imaging system was used to obtain images of protein bands.

### 2.7. Gene Silencing of FOXO3a with Small Interfering RNA

To inhibit *FOXO3a* expression, small interfering RNA (siRNA) was synthesized (Bioneer). The siFOXO3a-518 target sequence was 5′–GACAAACGGCUCACUCUGU-3′, the siFOXO3a-1774 target sequence was 5′-CUCAACGAGUGCGAACCUU-3′, and the siFOXO3a-1895 target sequence was 5′-GAUGCUGACGGGUUGGAUU-3′. Lipofectamine RNAi Reagent was used to transfect siRNA according to the manufacturer’s instructions (Life Technologies, Carlsbad, CA, USA). The IPEC-J2 Cells were incubated for 24 h after siRNA and DON treatments.

### 2.8. Immunofluorescence Staining of Cells

Cell monolayers on coverslips treated with DON were disposed of in 4% paraformaldehyde for 15 min at room temperature and then washed with PBS for 5 min. To begin, the IPEC-J2 was blocked for 1 h. Following that, Rabbit anti-FOXO3a IgG was treated in a 1:200 antibody solution and incubated in cells overnight at 4 °C. After 5 min of washing with PBS, goat anti-Rabbit (488) (Thermo Fisher Scientific, Carlsbad, CA, USA) was added at a ratio of 1:500 to an antibody solution and incubated in the dark for 1 h. Following that, coverslips were adhered to slides and stained with DAPI (Vector Laboratories, Burlingame, CA, USA). The photographs were taken with a fluorescence microscope.

### 2.9. Statistics

To determine the significance of the control and treatment groups, data analysis was performed using the general linear model (PROC-GLM) and *T*-test of SAS. The error bars represent the standard errors of triplicate analysis. In comparison to the control group, a significant difference in the treatment group was denoted by the following symbols: * *p* < 0.05, ** *p* < 0.01. The difference was evaluated using the Duncan multiple range tests.

## 3. Results

### 3.1. DON Decreased the Viability of IPEC-J2

**Figure 1:**
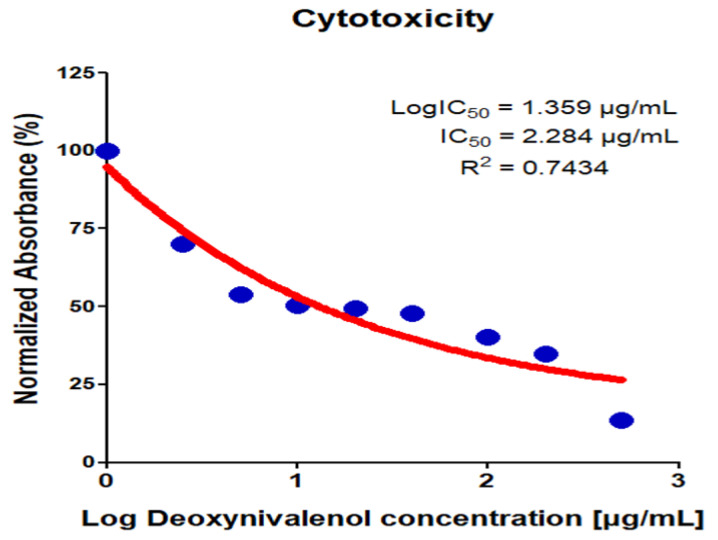
Deoxynivalenol decreased viability in IPEC-J2. Cell viability was determined by WST-1 assay. IPEC-J2 cells were incubated with different concentrations of DON (0.25, 0.5, 1, 2, 4, 10, 20, 50 μg/mL) for 24 h. Cell viability was expressed by the half-maximal inhibitory concentration (IC_50_).

To determine the viability of IPEC-J2 cells treated with DON, a WST-1 cell proliferation assay was used. IPEC-J2 cells were treated with varying concentrations of DON (0 μg/mL, 0.25 μg/mL, 0.5 μg/mL, 1 μg/mL, 2 μg/mL, 4 μg/mL, 10 μg/mL, 20 μg/mL, and 50 μg/mL) (Figure 1). The inhibitory concentration at half-maximum (IC50) was 2.284 μg/mL. In subsequent experiments, IPEC-J2 cells were treated with DON at a concentration of 2 μg/mL for 24 h to elucidate the molecular mechanism.

### 3.2. Identification and Validation of DEGs

**Figure 2:**
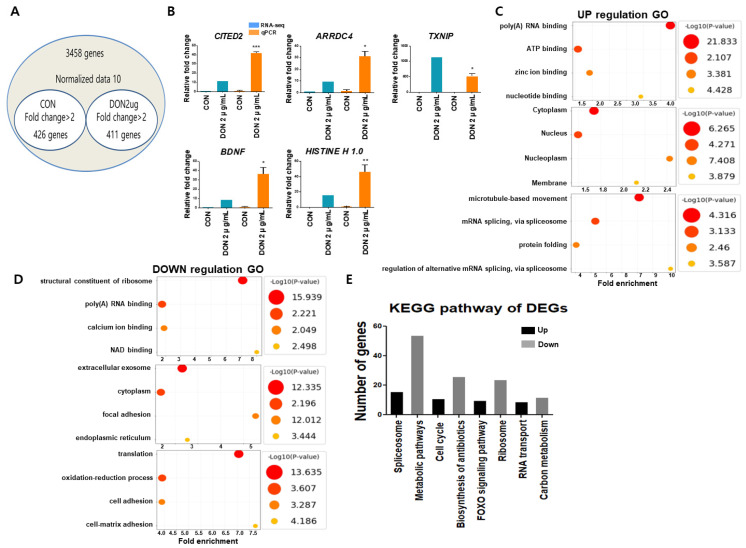
Gene-expression profiling in DON-treated porcine intestinal epithelial cells. (**A**) Venn diagram of genes up or downregulated 2-folds compared with control after DON treatment. (**B**) The TOP5 of upregulated differentially expressed genes (DEGs) data were validated by real-time quantitative PCR (*n* = 3). (**C**) Gene Ontology enrichment analysis of upregulated genes (*p* < 0.01). (**D**) Gene Ontology enrichment analysis of downregulated genes (*p* < 0.01). (**E**) Kyoto Encyclopedia of Genes and Genomes pathway classification enrichment analysis of differentially expressed genes (*p* < 0.05). * *p* < 0.05, ** *p* < 0.01, *** *p* < 0.001.

We identified 837 differentially expressed genes (DEGs) after analyzing the gene-expression profiles, including 411 upregulated and 426 downregulated genes (Figure 2A). Additionally, we confirmed the expression of the TOP 5 genes associated with upregulated DEGs using RT-PCR (Figure 2B). “Poly (A) RNA binding”, “ATP binding”, “zinc ion binding”, and “nucleotide binding” were included in the “molecular function” of upregulated DEGs identified through Gene Ontology (GO) analysis. The term “cellular component” was used interchangeably with “cytoplasm” and “nucleus.” The terms “microtubule-based movement” and “mRNA splicing via spliceosome” were used to describe “biological processes” (Figure 2C, Tables S1–S3). In comparison, the downregulated DEG demonstrated that the “molecular function” was associated with “structural constituent of the ribosome”, “poly (A) RNA binding”, “calcium ion binding”, and “NAD binding.” The terms “extracellular exosome”, “cytoplasm”, “focal adhesion”, and “cell surface” included the term “cellular component.” The terms “translation”, “oxidation-reduction process”, “cell adhesion”, “cell-matrix adhesion”, and “positive regulation of the apoptotic process” were used to describe “biological processes” (Figure 2D, Tables S4–S6). The major upregulated signaling pathways identified in the KEGG analysis were the “FOXO signaling pathway”, “MAPK signaling pathway”, and “p53 signaling pathway” (Figure 2E, Table S7). However, the major downregulated signaling pathways discussed include the metabolic pathway, antibiotic biosynthesis, and ribosome biosynthesis (Figure 2E, Table S8).

### 3.3. DON Induces Apoptosis in IPEC-J2

**Figure 3:**
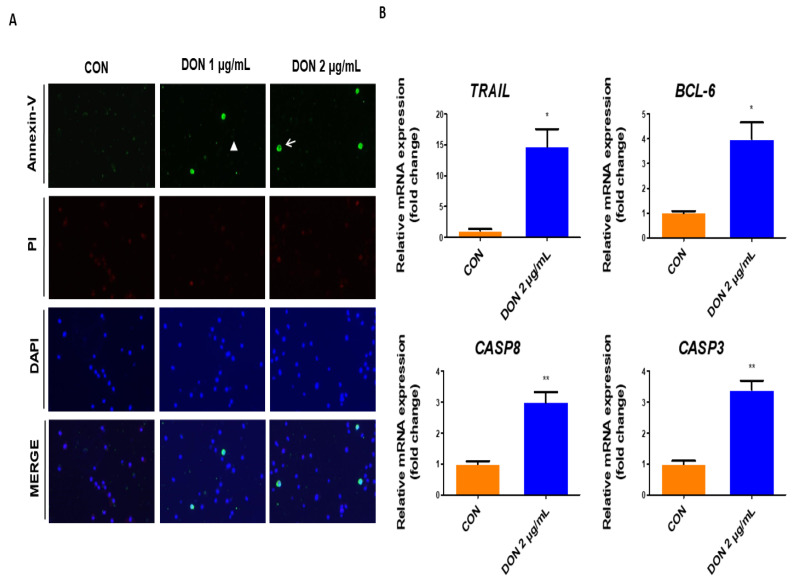
Deoxynivalenol induces apoptosis in porcine intestinal epithelial cells. (**A**) IPEC-J2 cells were treated at different concentrations of DON 1, 2 μg/mL for 24 h. Cell staining was performed through single-cell staining (Annexin-V; green, Propidium Iodide; red). Nuclei were stained with 4′,6-diamidino-2-phenylindole (DAPI; blue). (Scale bar means 20 μm.) (**B**) The mRNA levels of apoptosis-related genes (*TRAIL*, *BCL-6*, *CASP8*, *CASP3*) at DON 2 μg/mL concentration was compared with control (*n* = 3). Error bars indicated standard errors (SEs) of triplicate analysis. The arrow indicates Annexin-V positive-cell. The triangle indicates Annexin-V negative-cell. * *p* < 0.05, ** *p* < 0.01.

To determine whether DON induces apoptosis in IPECs, we performed double staining with Annexin-V and propidium iodide at various concentrations of DON (1 μg/mL, 2 μg/mL) (Figure 3A). Apoptosis was significantly increased in cells treated with DON 1, 2 μg/mL compared to untreated cells. Additionally, we confirmed the mRNA-expression level of apoptosis-related genes such as *TRAIL*, *BCL-6*, *CASP3*, and *CASP8*. These were significantly increased when compared to the untreated control (Figure 3B).

### 3.4. DON Triggers Translocation of FOXO3a to the Nucleus

**Figure 4:**
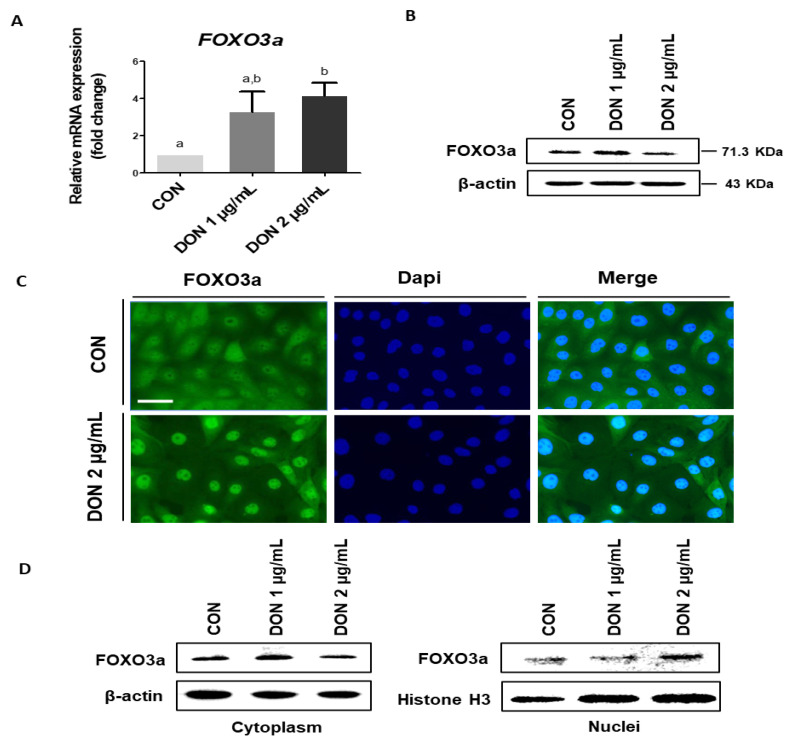
Deoxynivalenol mediates *FOXO3a* expression and leads to translocation. (**A**,**B**) The expression levels of *FOXO3a* on the different concentrations of DON 1, 2 μg/mL were compared with the control (*n* = 3). Error bars indicated standard errors (SEs) of triplicate analysis. Significant differences between control and treatment groups are indicated as a,b. (**C**,**D**) *FOXO3a* translocation was evaluated using Western blotting and immunocytochemistry. Nuclei were stained with 4′,6-diamidino-2-phenylindole (DAPI; blue). (Scale bar means 40 μm).

We used quantitative real-time PCR, Western blotting, and immunocytochemistry to determine the expression of *FOXO3a*. At 1 ug/mL DON, the level of *FOXO3a* mRNA expression was not significantly different from the control. However, when DON 2 μg/mL was used, the level of *FOXO3a* mRNA expression was significantly increased compared to the control (Figure 4A). There was no difference in the level of *FOXO3a* protein expression (Figure 4B). Immunocytochemistry analysis revealed that DON induces *FOXO3a* nuclear translocation (Figure 4C). Additionally, at a DON 2 μg/mL concentration, *FOXO3a* expression was significantly decreased in the cytoplasm and significantly increased in the nucleus (Figure 4D). Following these findings, subsequent experiments were conducted at a DON concentration of 2 μg/mL.

### 3.5. Knockdown of FOXO3a Reduces Apoptosis in Porcine Intestinal Epithelial Cells

**Figure 5:**
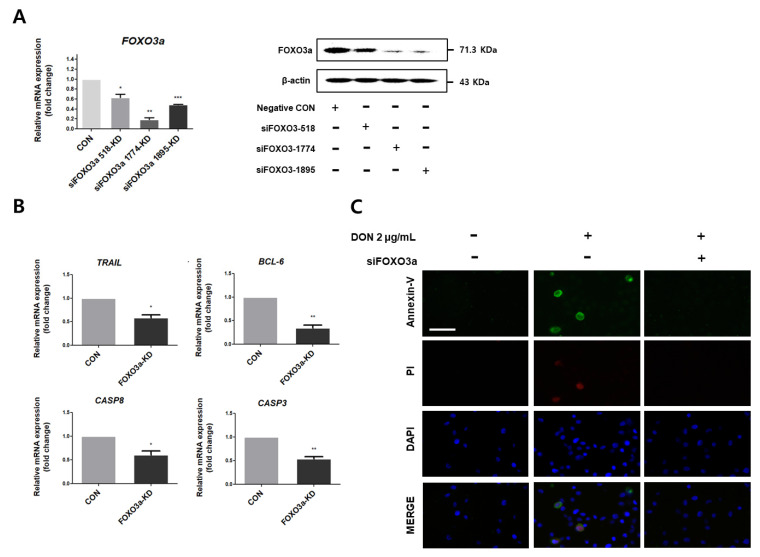
Knockdown of *FOXO3a* attenuates apoptosis. (**A**) After siFOXO3a treatment, levels of *FOXO3a* protein expression and mRNA expression. (**B**) After DON and siFOXO3a treatment, mRNA-expression level of apoptosis-related genes (*TRAIL*, *BCL-6*, *CASP3*, and *CASP8*) were compared with the negative control. (**C**) Apoptosis analysis was performed through single-cell staining using the Annexin-V (green) and propidium (red) iodide. Nuclei were stained with 4′,6-diamidino-2-phenylindole (DAPI; blue). (Scale bar means 40 μm). * *p* < 0.05, ** *p* < 0.01, *** *p* < 0.001.

We investigated whether silencing *FOXO3a* affects apoptosis. We confirmed the levels of mRNA and protein expression in three distinct sequences using quantitative real-time PCR and Western blotting (Figure 5A). In light of these findings, we proceeded with subsequent experiments using siFOXO3a-1774. After treatment with siFOXO3a, we analyzed the mRNA-expression levels of apoptosis-related genes (*TRAIL*, *BCL-6*, *CASP3*, and *CASP8*). These results were significantly reduced when compared to the control group (Figure 5B). Additionally, when cells were treated with siRNA, apoptosis was significantly reduced (Figure 5C).

### 3.6. Inhibition of ERK1/2 Represses Apoptosis and Translocation of FOXO3a

**Figure 6:**
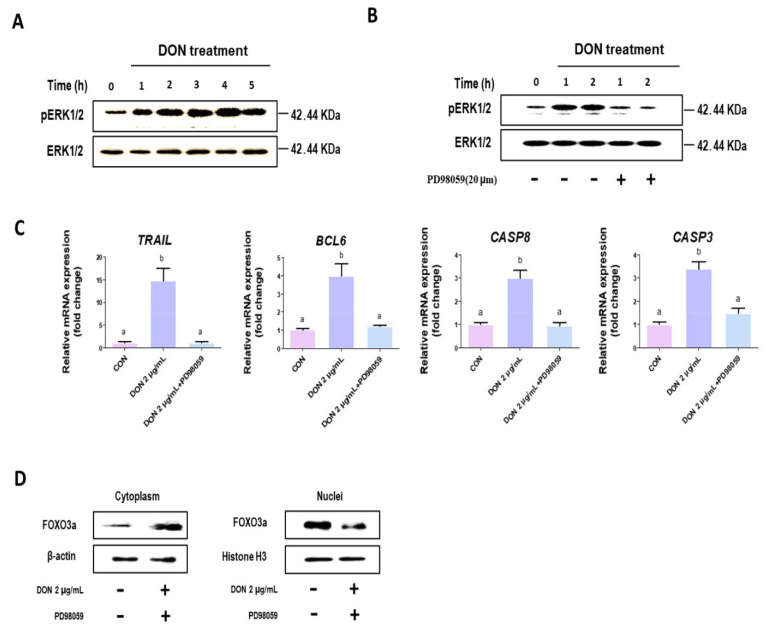
Doexynivalenol increased ERK1/2 phosphorylation, and inhibition of ERK1/2 suppresses a translocation of FOXO3a. (**A**) ERK1/2 phosphorylation level in IPEC-J2 cells after DON treatment in concentrations of 2 μg/mL for (1 h, 2 h, 3 h, 4 h, 5 h). (**B**) Level of phosphorylation (0 h, 1 h, and 2 h) of ERK1/2 by PD98059 in cells treated with DON at a concentration of 2 μg/mL. (**C**) The mRNA-expression levels of apoptosis-related genes (*TRAIL*, *BCL-6*, *CASP3*, and *CASP8*) on PD98059-treated IPEC-J2. (**D**) Western blotting analysis show inhibition of *FOXO3a* translocate after PD98059 treatment. Lowercase letters (a and b) mean significant differences between groups based on Duncan’s multi-range test.

On IPEC-J2 treated with DON, we examine the phosphorylation of ERK1/2. DON stimulated ERK1/2 phosphorylation in a time-dependent manner (1 h, 2 h, 3 h, 4 h, and 5 h) (Figure 6A), whereas ERK1/2 inhibitor PD98059 inhibited ERK1/2 phosphorylation (Figure 6B). Additionally, inhibition of ERK1/2 phosphorylation decreased the expression of genes associated with apoptosis when compared to untreated control (Figure 6C). Following that, we examined the effect of inhibiting ERK1/2 phosphorylation on *FOXO3a* translocation. When cells were treated with PD98059 and DON, the level of *FOXO3a* protein expression in the cytoplasm was increased compared to untreated controls, whereas when compared to untreated controls, the level of *FOXO3a* protein expression in the nucleus was decreased (Figure 6D).

## 4. Discussion

*Fusarium* produces DON during the growth and storage of grains. DON-contaminated feed is found worldwide and is also referred to as vomitoxin since high concentrations cause vomiting [25]. Consumption of feed contaminated with DON results in decreased feed intake, delayed growth, and weight loss [26,27]. Additionally, DON alters the intestine’s morphology and impairs nutrient absorption [28]. As a result of ingesting DON, livestock productivity can be reduced, resulting in economic loss. Porcine is one of the most susceptible species to DON, which is associated with a high grain intake [29,30]. At the cellular level, DON toxicity causes mitochondrial dysfunction, which results in the accumulation of reactive oxygen species (ROS), apoptosis, and inflammation, as well as inhibits protein synthesis via binding to the ribosome [31,32]. However, the effect of DON on porcine intestinal epithelial cells is unknown. As a result, we profiled the gene expression of IPEC-J2 cells following DON treatment. Our study sought to confirm DON-induced apoptosis in porcine intestinal epithelial cells via the FOXO3a-signaling pathway.

The molecular mechanism by which DON induces IPEC-J2 requires additional research. As a result, we profiled the expression of 837 DEGs in porcine intestinal epithelial cells. Cell adhesion and cell-matrix adhesion were identified as the main GO terms, and the FOXO-signaling pathway, MAPK-signaling pathway, and p53-signaling pathway were identified as the primary pathways. *FOXO3a*, a member of the Forkhead transcription factor *FOXO* subfamily, has been implicated in processes such as apoptosis, metabolism, stress response, and proliferation [33]. *FOXO3a*, in particular, acts as an apoptosis regulator [34]. As a result of these findings, we hypothesized that DON could induce apoptosis via the FOXO-signaling pathway.

To confirm DON-induced apoptosis in the small intestine, we performed real-time PCR analysis on the expression of apoptotic genes. *FOXO3a* has been known to regulate the expression of genes involved in cell death, including tumor-necrosis-factor-related apoptosis-inducing ligand (*TRAIL*), Bcl-2 interacting mediator of cell death (*BIM*), B-cell CLL/lymphoma 6 (*BCL-6*), and Fas ligand (*FASLG*) [35]. *TRAIL* induces apoptosis by activating death receptors, which activate *caspase-8* and result in apoptosis. *BCL-6* indirectly inhibits the pro-survival BCL-2 family, resulting in an increase in mitochondrial permeability and activation of caspase-3, which induces apoptosis [36]. Our study established that DON induces apoptosis in IPEC-J2 cells via apoptosis associated with genes such as *TRAIL*, *BCL-6*, *CASP3*, and *CASP8* and that DON induces the translocation of *FOXO3a* into the nucleus. Numerous additional studies indicate that DON induces apoptosis in porcine intestinal epithelial cells via diverse pathway [13,21,37]. However, studies of DON-induced apoptosis via FOXO3a-signaling pathway do not yet exist.

Additionally, we confirmed that DON-induced ERK1/2 phosphorylation. Additionally, to determine whether DON translocates *FOXO3a* to the nucleus and induces apoptosis, we used siRNA and the ERK1/2 inhibitor PD98059 to induce *FOXO3a* knockdown. ERK1/2 are protein serin/threonine kinase and become involved in the Ras-Raf-MEK-ERK signal-transduction cascade that is related to various processes such as cell cycle, cell adhesion, migration, differentiation, metabolism, proliferation, transcription, and cell survival. ERK1/2 cascade is also associated with *FOXO*, which regulates apoptosis and cell cycle arrest [38,39]. Protein kinase B (PKB), ERK, serum- and glucocorticoid-inducible kinases, and IǩB kinase isoform β were previously identified as regulating *FOXO3a* translocation [40]. The phosphorylation of ERK1/2 by DON has been shown in previous studies [41]. Our study established that DON induces ERK1/2 in a time-dependent manner. Following that, when IPEC-J2 cells were treated with the PD98059 and DON, the treatment decreased the expression of apoptosis-related genes and inhibited the translocation of *FOXO3a* to the nucleus. Recent research indicates that the silencing of *FOXO3a* inhibited apoptosis in human gastric mucosa cell lines treated with DON [42]. We examined the effect of *FOXO3a* silencing on DON-induced apoptosis. This finding demonstrates that DON can induce apoptosis in porcine intestinal epithelial cells via the FOXO3a-signaling pathway.

**Figure 7:**
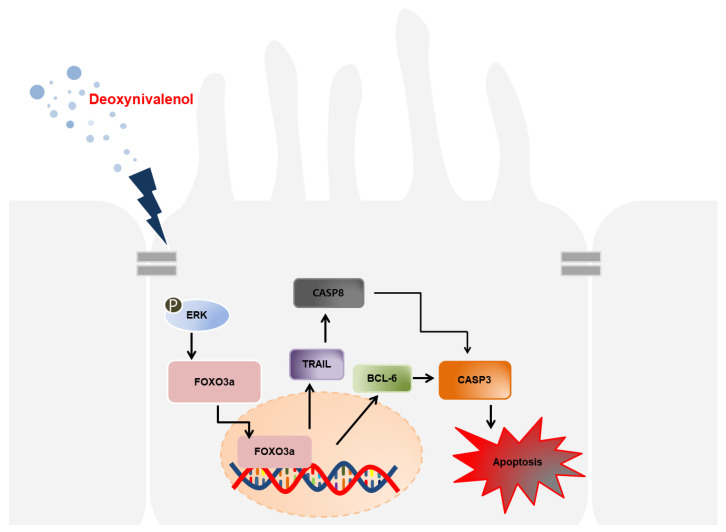
Schematic illustrating of current working hypothesis toward DON-mediated apoptosis via the FOXO3a-signaling pathway. DON-induced translocation of *FOXO3a* to nucleus, which regulated *TRAIL* and *BCL-6* expression and lead to activation of *CAPS8* and *CASP3*. Finally, *CASP8*- and *CASP3*-induced apoptosis on porcine intestinal epithelial cells via the ERK1/2-signaling pathway.

These results established that DON can induce apoptosis in porcine intestinal epithelial cells via the FOXO3a-signaling pathway (Figure 7). This study demonstrated that DON-contaminated feedstuffs can deteriorate pig small-intestine health, resulting in decreased productivity.

## Figures and Tables

**Table 1:** List of PCR primers.

Genes	Description	Accession No.		Sequence (5′–3′)
TRAIL	Tumor-necrosis-factor-related apoptosis-inducing ligand	NM_001024696	Forward	GCA GAC CTG TGT GTT GAT CC
			Reverse	GGG ATC CCA GAA ACT GTC AT
BCL-6	B-cell CLL/lymphoma 6	XM_005657112	Forward	GTG TCC TAC GGT GCC TTT TT
			Reverse	TGA CGC AGA ATG TGA TGA GA
FOXO3	Fork headbox O3	NM_001135959	Forward	TCA GCC AGT CTA TGC AAA CC
			Reverse	CCA TGA GTT CGC TAC GGA TA
CASP3	Caspase3	NM_214131	Forward	CTC AGG GAG ACC TTC ACA AC
			Reverse	GCA CGC AAA TAA AAC TGC TC
CASP8	Caspase8	NM_001031779	Forward	GCC GAC TGG ATG TGA TTT TAT
			Reverse	TGT TTC TGA GGT TGC TGG TC
GAPDH	Glyceraldehyde-3-phosphate dehydrogenase	NM_001206359	Forward	ACA CCG AGC ATC TCC TGA CT
			Reverse	GAC GAG GCA GGT CTC CCT AA

## Data Availability

Not applicable.

## Supplementary Materials

The following are available online at https://www.mdpi.com/article/10.3390/toxics10090535/s1, Additional information about Gene Ontology and KEGG pathway analysis to differentially expressed genes (PDF), Table S1: biological process of upregulated differentially expressed genes, Table S2: cellular component of upregulated differentially expressed genes, Table S3: molecular function of upregulated differentially expressed genes, Table S4: biological process of downregulated differentially expressed genes, Table S5: cellular component of downregulated differentially expressed genes, Table S6: molecular function of downregulated differentially expressed genes, Table S7: KEGG pathway of upregulated differentially expressed genes, Table S8: KEGG pathway of downregulated differentially expressed genes.
