# Supplementary material for: Deoxynivalenol Induces Apoptosis via FOXO3a-Signaling Pathway in Small-Intestinal Cells in Pig
Source: Toxics. 2022 Sep 13;10(9):535. doi: 10.3390/toxics10090535 (PMC9503759; doi:10.3390/toxics10090535)
Supplement: Supplementary file 1 [file toxics-10-00535-s001.zip › toxics-1877197-SI.pdf]

**Supplemental Table S1. Biological process of upregulated differentially expressed genes**

| Term | Count | PValue | Genes |
|---|---|---|---|
| microtubule-based movement | 8 | 4.83E-05 | CENPE, KIF18A, DYNC1LI2, KIF5B, KIF21A, KIF20A, KIF11, KIF20B |
| mRNA splicing, via spliceosome | 8 | 0.000737 | SNW1, SYF2, TRA2B, METTL3, TRA2A, CWC22, PRPF40A, BCAS2 |
| protein folding | 8 | 0.003469 | DNAJC1, PPWD1, PFDN2, CCT8, PPIG, CWC27, PPIL4, PDRG1 |
| regulation of alternative mRNA splicing, via spliceosome | 6 | 0.000259 | RBM25, SF3A1, RBM8A, TRA2B, NSRP1, RBM5 |
| mRNA processing | 6 | 0.000367 | SON, RBM8A, AKAP8L, RBBP6, SMNDC1, QKI |
| double-strand break repair via homologous recombination | 6 | 0.001674 | RAD50, WDR48, RBBP8, NABP2, RAD54L, SMC5 |
| nucleosome assembly | 6 | 0.005805 | BRD2, LOC100510904, LOC100516295, LOC100738859, SMARCA5, RSF1 |
| stem cell population maintenance | 5 | 0.006131 | DDX6, MED10, NKAP, SMC3, EIF4E |
| mitotic chromosome condensation | 4 | 0.001099 | AKAP8L, AKAP8, NCAPG, SMC4 |
| positive regulation of transforming growth factor beta receptor signaling pathway | 4 | 0.002495 | STK11, SNW1, CITED2, THBS1 |
| positive regulation of ubiquitin-protein transferase activity | 4 | 0.003125 | ARRDC4, TRIB3, BMI1, TOPORS |
| intrinsic apoptotic signaling pathway in response to DNA damage by p53 class mediator | 4 | 0.007675 | SNW1, EP300, AEN, TOPORS |

## Supplemental Table S2. Cellular component of upregulated differentially expressed genes

| Term | Count | PValue | Genes |
|---|---|---|---|
| cytoplasm | 77 | 5.43E-07 | TES, AKAP8L, NKAP, ADM, PHAX, KIF11, SMC3, CHD1, SMC4, ETS2, PHTF1, NUDT4, PTTG1, EME1, PCF11, DPF2, EP300, DLGAP5, IER2, CEP68, BRD2, USP47, TSC22D2, PRPF40A, PPP4R3B, WDR35, LRRC41, ENAH, KCTD9, DMTF1, DDIT3, FAM185A, BIRC5, TERF2IP, TXNIP, PPIG, SGK1, ERGIC2, SLU7, GTF2A1, GGNBP2, CDCA2, RBM8A, SRF, NCAPG, CCNB1, UBB, FLRT2, UBC, MBIP, FLNC, SMNDC1, SOCS7, EIF4E, ATF7IP, TSNAX, NOP58, MAP3K1, GADD45B, BDNF, FUS, PLK1, RDX, UPF3B, EIF2S1, QKI, CENPE, SNRNP40, KIF18A, CDK7, NABP2, RLIM, ID3, PPIL4, ZCCHC2, CDKN3, METAP2 |
| nucleus | 69 | 5.36E-05 | LTV1, WDR48, TES, CITED2, CCNF, ARID4A, PHAX, BMI1, CHD1, ETS2, NUDT4, PSMD8, STK11, PTTG1, CREB3L1, TLK2, PHACTR1, DLGAP5, CEP68, BRD2, USP47, PRKCI, OSR1, TSC22D2, ARID5B, LRRC41, RHOB, PHF20L1, DNAJC1, DMTF1, MORF4L2, CRY2, BIRC5, SRFBP1, TRIB3, KIF20A, SGK1, ZMYND11, VGLL3, GGNBP2, LOC100510904, BAZ2B, CCNB2, CCNB1, PABPN1, UBB, UBC, RBBP8, SAFB2, RBBP6, SMNDC1, ZKSCAN1, TSNAX, AKIRIN2, POU2F1, GADD45B, GADD45A, CCDC59, DONSON, EIF2S1, PHC3, QKI, PNRC2, CENPE, KIF18A, ID3, ESF1, CALM2, CDKN3 |
| nucleoplasm | 46 | 3.91E-08 | CDCA2, PPP1R10, BOD1L1, AKAP8, NKAP, HNRNPU, PHAX, SMC3, SMC4, CLINT1, ETS2, EXOSC9, DPF2, SAFB2, RAD54L, UBXN7, RBBP6, CCT8, RBM5, IER2, ATF7IP, USP8, TIA1, POU2F1, FUS, PRPF40A, AEN, PPP4R3B, MRPL46, PHC3, SGO2, SNRNP40, RAD50, RYBP, DMTF1, FUBP1, WAC, NABP2, RLIM, LUC7L3, PFDN2, PPIG, KIF20B, SGK1, ZMYND11, PPIL4 |
| membrane | 31 | 1.32E-04 | DIRAS3, DDX6, AKAP8, HNRNPU, NCAPG, HMMR, KIF11, CLINT1, CCNB2, CCNB1, STK11, KIF5B, NOP58, PRRC2C, PRPF40A, DDX52, GNL2, MED4, EIF2S1, LRRC41, HCFC1, HNRNPM, CENPE, RAD50, DYNC1LI2, DRG1, OCIAD1, DCP1A, ERGIC2, SLU7, EIF3A |
| nucleolus | 28 | 6.96E-07 | LOC100516295, PHTF1, MTDH, RPF1, EXOSC9, TRA2A, MBIP, BRIX1, LLPH, RBM34, RAB8A, NGDN, PLK1, SMARCA5, DNTTIP2, UPF3B, AEN, DDX52, GNL2, FTSJ3, EWSR1, MORF4L2, RPL7L1, FRG1, PFDN2, KIF20B, ERGIC2, EIF3A |
| centrosome | 19 | 3.13E-06 | CEP68, PLK1, CKAP2, NCAPG, PHAX, PPP4R3B, WDR35, AURKA, IFT20, CCNB2, DYNC1LI2, CCNB1, DPF2, TRIM59, CCT8, KIF20B, CALM2, RAB8A, PAFAH1B1 |
| catalytic step 2 spliceosome | 13 | 3.88E-09 | SF3A1, RBM8A, HNRNPU, CWC27, CDC40, HNRNPM, SNRNP40, SNW1, SYF2, CWC22, FRG1, SLU7, BCAS2 |
| nuclear speck | 9 | 1.34E-04 | RBM25, SON, RBM8A, METTL3, AKAP8L, CWC22, NSRP1, SLU7, TOPORS |
| kinesin complex | 7 | 1.14E-04 | CENPE, KIF18A, KIF5B, KIF21A, KIF20A, KIF11, KIF20B |
| nuclear matrix | 7 | 2.30E-04 | HNRNPM, SNW1, CFL2, AKAP8L, AKAP8, PRPF40A, SMC3 |
| spindle pole | 6 | 0.001803 | CCNB1, PLK1, KIF11, SMC3, CALM2, TOPORS |
| spindle microtubule | 5 | 0.001832 | PLK1, BIRC5, KIF11, CALM2, AURKA |
| small-subunit processome | 5 | 0.004001 | NOP56, UTP6, NOP58, NGDN, MPHOSPH10 |
| condensed chromosome | 4 | 0.003261 | AKAP8, SMARCA5, NCAPG, BAZ1B |
| precatalytic spliceosome | 4 | 0.008703 | PRPF38B, SNRNP40, SON, BCAS2 |
| mitotic spindle midzone | 3 | 0.005988 | CENPE, KIF18A, KIF20B |

## Supplemental Table S3. Molecular function of upregulated differentially expressed genes

| Term | Count | PValue | Genes |
|---|---|---|---|
| poly(A) RNA binding | 61 | 1.47E-22 | TES, PPP1R10, AKAP8L, NKAP, HNRNPU, FAM208A, THUMPD1, MRPL32, RBM34, DNTTIP2, PRPF40A, DDX52, GNL2, CDC40, FTSJ3, EWSR1, SYF2, RPL7L1, FRG1, SRFBP1, PPIG, DDX6, MRPS35, LOC100516295, AKAP8, MRPS30, MTDH, RPF1, PABPN1, EXOSC9, UBC, TRA2A, SAFB2, BRIX1, RBBP6, LLPH, SMNDC1, EIF4E, TRMT1L, NOP58, PRPF38B, TIA1, FUS, FAM46A, CCDC59, RDX, PRRC2C, RSRC2, EIF2S1, ARCN1, HNRNPM, SNRNP40, SON, SNW1, CMSS1, FUBP1, TRMT6, MPHOSPH10, ESF1, PPIL4, METAP2 |
| ATP binding | 34 | 0.007809 | DDX6, ROCK1, DDX1, TWF1, KIF11, SMC3, SMC4, CHD1, AURKA, STK11, KIF5B, CHORDC1, TLK2, RAD54L, ABL2, KIF21A, CCT8, PMS1, DDX18, PRKCI, MAP3K1, HSPA4, PLK1, SMARCA5, DDX52, CENPE, KIF18A, CDK7, DNAJA2, TRIB3, KIF20A, KIF20B, SGK1, CFTR |
| zinc ion binding | 33 | 4.16E-04 | PHF3, TES, JADE1, RSF1, BAZ2B, PRICKLE1, BMI1, RBM4, UBR5, CHORDC1, DPF2, EP300, RBBP6, LONRF3, RBM5, MORC3, MAP3K1, FUS, CNBP, SREK1IP1, BAZ1B, PHC3, TOPORS, PHF20L1, EWSR1, G2E3, RLIM, BIRC5, TRIM59, ZMYND11, CBLL1, SLU7, ZCCHC2 |
| nucleotide binding | 17 | 3.73E-05 | RBM27, RBM25, TIA1, RBM8A, NIFK, FUS, UPF3B, HNRNPM, RBM4, PABPN1, EWSR1, TRA2B, TRA2A, SAFB2, RBM34, PPIL4, RBM5 |
| RNA binding | 13 | 7.28E-04 | DDX18, SF3A1, PRPF40A, THUMPD1, QKI, SON, EWSR1, FUBP1, UBR5, CWC22, BRIX1, SMNDC1, EIF4G2 |
| mRNA binding | 11 | 2.96E-06 | RBM25, RBM8A, PCF11, TRA2B, LUC7L3, UPF3B, DCP1A, NSRP1, QKI, EIF3A, RBM5 |
| ATPase activity | 7 | 0.005128 | ATF7IP, CENPE, SMARCA5, KIF21A, KIF20A, KIF20B, PMS1 |
| microtubule motor activity | 6 | 0.002196 | CENPE, DYNC1LI2, KIF5B, KIF21A, KIF20A, KIF20B |
| ATP-dependent microtubule motor activity, plus-end-directed | 4 | 0.001059 | KIF18A, KIF5B, KIF11, KIF20B |

**Supplemental Table S4. Biological process of downregulated differentially expressed genes**

| Term | Count | PValue | Genes |
|---|---|---|---|
| translation | 26 | 2.32E-14 | SLC25A1, RPL3, RPL10, RPL12, RPL8, RPS4X, RPS14, RPL18A, RPL13, RPS11, RPL18, RPL39, SLC25A24, RPS9, RPS5, LOC100623540, RPSA, EEF2, MRPS6, RPS27, RPS29, RPL27A, ATM, RPS20, SLC25A5, SLC25A6 |
| oxidation-reduction process | 12 | 2.47E-04 | LDHA, SH3PXD2A, GPX4, SCD, FTH1, ME1, CYBA, MSMO1, TXN, PGD, GAPDH, CYP51 |
| cell adhesion | 11 | 5.16E-04 | FERMT1, TINAGL1, NOV, ITGA2, CD9, ITGA6, ITGAV, IGFBP7, RPSA, ATP1B1, CTGF |
| cell-matrix adhesion | 8 | 6.51E-05 | BCAM, ITGB4, ITGA2, EMP2, ITGA6, ITGAV, NID2, CTGF |
| positive regulation of apoptotic process | 8 | 0.006305545 | BNIP3L, BCL2L11, ITGA6, APBB2, ATG7, DUSP6, BCL2L1, TGM2 |
| glycolytic process | 6 | 6.31E-05 | GPI, TPI1, PGK1, ENO1, GAPDH, HK1 |
| cytoplasmic translation | 5 | 0.001542102 | RPLP1, RPLP2, RPL15, RPL8, RPL29 |
| cellular response to oxidative stress | 5 | 0.002470359 | PARP1, LONP1, TXN, NFE2L1, SLC25A24 |
| translational elongation | 4 | 2.10E-04 | EEF1G, RPLP1, RPLP2, EEF2 |
| ribosomal small subunit assembly | 4 | 0.003343141 | RPS27, RPS5, RPSA, RPS10 |
| cell adhesion mediated by integrin | 3 | 0.009696745 | NOV, ITGA2, ITGA6 |

**Supplemental Table S5. Cellular component of downregulated differentially expressed genes**

| Term | Count | PValue | Genes |
|---|---|---|---|
| extracellular exosome | 80 | 4.62E-13 | SLC25A1, LAD1, GPI, TINAGL1, RPL3, SLC44A1, ITGB4, RPLP1, DBI, ENO1, ACTB, ACTG1, RPS4X, CDH6, LGALS1, LAMP1, PLAU, RPLP2, TIMP3, SH3BGRL2, ITGAV, RPS11, GLUL, RPS10, SPTAN1, TGM2, BCAS1, RPS9, TPI1, RPS5, TUBB, ANO6, MIF, ATP1B1, PGD, SDCBP2, EEF1G, BCAM, PKM, GPRC5A, PSME1, S100A4, TAGLN2, TNIK, PABPC1, SLC25A5, CD46, B4GALT5, CSTB, GRN, AHCY, MVK, MGST3, RPL12, TACSTD2, TXN, NID2, NDRG1, PPL, ACACA, ACAT2, LDHA, IGFBP7, SFN, ST3GAL1, VAT1, ST14, GALNT2, GOLM1, IDH1, MSN, BAIAP2, EEF2, PTPN13, DAB2, KRT19, CALM3, RPS20, CALR, ITM2B |
| cytoplasm | 64 | 0.006371148 | GPI, VEZT, TINAGL1, ACY1, YBX3, TMEM189, CCND2, FTH1, GRB10, TNS3, TNPO3, APOBEC1, SREBF1, SERPINB1, CXADR, PARP1, HMGCS1, MIF, PGD, SDCBP2, DUSP6, EEF1G, ACLY, TUBB2B, NOV, ELMO1, PSME1, S100A6, SELENOW, TNIK, PABPC1, GAPDH, CSNK1G2, MT1D, FTL, CSTB, ZNF395, AHCY, ACSS2, TXN, NDRG1, NPAS2, LDHA, GRK5, MT-2B, PGK1, SMYD2, EIF4EBP2, SFN, APBB2, METTL8, ATG7, IVNS1ABP, FDPS, SRD5A1, IRX3, MACC1, MSN, EEF2, PTPN13, SSH1, PTPRE, FABP3, BCL2L1 |
| focal adhesion | 28 | 9.72E-13 | RPL3, RPL12, RPLP1, HACD3, RPL8, ACTB, ACTG1, RPS4X, GRK5, PLAU, RPLP2, ITGAV, RPS11, RPL18, RPS1 , TNS3, TGM2, RPS9, RPS5, ITGA2, HMGA1, MSN, FERMT1, DAB2, ITGA6, PABPC1, CALR, CD46 |
| endoplasmic reticulum | 20 | 3.60E-04 | TPD52, CDS1, USP25, BNIP3L, MGST3, SSR2, BNIP3, ATP10A, ELOVL6, DHCR24, DBI, SGPP2, CYP51, EEF1B2 EEF1G, EBP, RCN1, P4HA2, FDFT1, TGM2 |
| cell surface | 16 | 5.07E-04 | IGSF5, CXADR, ITGB4, F2R, EMP2, ANO6, MIF, MST1R, BCAM, PLAU, CD109, LIPG, ITGAV, ADAM8, CD46 LDLR |
| cytosolic large ribosomal subunit | 12 | 2.16E-08 | RPL3, RPL10, RPL18A, RPL27A, RPL12, RPLP1, RPLP2, RPL13, RPL15, RPL8, RPL29, RPL18 |
| ribosome | 10 | 3.47E-07 | RPS14, RPS29, RPL27A, LOC100623540, RPLP2, ATM, RPL8, RPL18, RPS10, RPL39 |
| myelin sheath | 10 | 8.98E-04 | GPI, PKM, MSN, MIF, SLC25A5, ATP1B1, GLUL, NDRG1, ACTB, ACTG1 |
| cytosolic small ribosomal subunit | 9 | 1.55E-06 | RPS4X, RPS9, RPS27, RPS29, RPS5, RPS20, RPSA, RPS11, RPS10 |
| nuclear envelope | 6 | 0.008395067 | BNIP3L, PARP1, BNIP3, MGST3, SIGMAR1, S100A6 |
| polysome | 5 | 0.001397223 | RPS4X, RPL10, CALR, EEF2, YBX3 |
| integrin complex | 4 | 0.004615113 | ITGB4, ITGA2, ITGA6, ITGAV |

**Supplemental Table S6. Molecular function of downregulated differentially expressed genes**

| Term | Count | PValue | Genes |
| --- | --- | --- | --- |
| structural constituent of ribosome | 30 | 1.15E-16 | SLC25A1, RPL3, RPL10, RPL12, RPLP1, RPL8, RPS4X, RPS14, RPL18A, RPLP2, RPL13, RPL15, RPS11, RPL18, RPS10, RPL39, SLC25A24, RPS9, RPS5, LOC100623540, RPSA, MRPS6, RPS27, RPS29, RPL27A, ATM, RPS20, RPL29, SLC25A5, SLC25A6 |
| poly(A) RNA binding | 26 | 0.00600838 | CSTB, GRN, POP7, RPL3, RPL12, TXN, ENO1, RPL8, RPS4X, LGALS1, RPL18A, RPL13, RPS11, RPS10, FDPS, RPS9, PARP1, PABPC4, EEF2, RPS27, PKM, RPS20, S100A4, PABPC1, RPL29, SLC25A5 |
| calcium ion binding | 20 | 0.008940336 | TPD52, S100A2, DGKA, CETN2, ITPR3, LRP8, NID2, SYTL2, CDH6, RCN1, PRRG1, S100A6, CHP1, CALM3, ADAM8, S100A4, CALR, LDLR, SPTAN1, SLC25A24 |
| NAD binding | 5 | 0.003178343 | AHCY, PARP1, IDH1, ME1, GAPDH |

**Supplemental Table S7. KEGG pathway of upregulated differentially expressed genes**

| Term | Count | PValue | Genes |
|---|---|---|---|
| Spliceosome | 16 | 6.16187E-09 | RBM25 PRPF38B SF3A1 RBM8A HNRNPU PRPF40A CDC40 HNRNPM SNRNP40 SNW1 SYF2 TRA2B TRA2A SLU7 SMNDC1 BCAS2 |
| Cell cycle | 11 | 3.27472E-05 | CCNB2 CDK7 CCNB1 TGFB2 PTTG1 GADD45B GADD45A RAD21 PLK1 EP300 SMC3 |
| FoxO signaling pathway | 10 | 0.000551038 | CCNB2 STK11 CCNB1 TGFB2 USP7 GADD45B GADD45A PLK1 EP300 SGK1 |
| RNA transport | 9 | 0.005401042 | FXR1 RBM8A EIF3J UPF3B PHAX EIF4E EIF2S1 EIF3A EIF4G2 |
| Oocyte meiosis | 7 | 0.012570634 | CCNB2 PPP3CB PTTG1 PLK1 SMC CALM2 AURKA |
| Ribosome biogenesis in eukaryotes | 6 | 0.014823538 | NOP56 UTP6 NOP58 MPHOSPH10 GNL2 WDR43 |
| MAPK signaling pathway | 10 | 0.028731975 | TGFB2 PPP3CB MAP3K1 GADD45B BDNF GADD45A DDIT3 SRF FLNC LAMTOR3 |
| Vasopressin-regulated water reabsorption | 4 | 0.037725291 | DYNC1LI2 CREB3L1 DYNLL1 RAB5A |
| p53 signaling pathway | 5 | 0.038328943 | CCNB2 CCNB1 GADD45B GADD45A THBS1 |
| TGF-beta signaling pathway | 5 | 0.047175706 | TGFB2 ROCK1 EP300 ID3 THBS1 |

## Supplemental Table S8. KEGG pathway of downregulated differentially expressed genes

| Term | Count | PValue | Genes |
|---|---|---|---|
| Metabolic pathways | 54 | 4.97E-05 | GPI PGAP1 ACY1 GALNT18 DGKA MSMO1 SAT1 HK1 LIPG ME1 HADH GLUL TPI1 HMGCS1 PGD LSS ACLY NDUFS8 PKM NME7 B3GNT3 PGP POLR1D ND1 GAPDH ND4 CDS1 AHCY MVK ACSS2 AK4 ACACA ACAT2 POLD3 LDHA EBP PGK1 SC5D COX1 LOC100524239 ST3GAL1 FDFT1 GALNT7 FDPS RRM2 GALNT2 IDH1 MBOAT2 DHCR24 CYP51 SQLE P4HA2 FASN LPIN1 |
| Biosynthesis of antibiotics | 26 | 3.90E-11 | GPI MVK ACSS2 ACY1 MSMO1 AK4 ACAT2 HK1 LDHA PGK1 SC5D HADH FDFT1 FDPS TPI1 HMGCS1 IDH1 PGD LSS CYP51 ACLY SQLE PKM NME7 PGP GAPDH |
| Ribosome | 24 | 2.83E-12 | RPS9 RPL3 RPL10 RPS5 RPL12 RPLP1 LOC100623540 RPSA RPL8 RPS4X RPS27 RPL18A RPS29 RPL27A RPLP2 RPL13 RPS20 RPL15 RPS2 RPS11 RPL29 RPL18 RPS10 RPL39 |
| Carbon metabolism | 12 | 9.96E-05 | GPI PKM ACSS2 TPI1 IDH1 ME1 PGP PGK1 PGD GAPDH ACAT2 HK1 |
| Proteoglycans in cancer | 12 | 0.013996961 | PLAU ITGA2 IGF2 TIMP3 MSN ITGAV ITPR3 CAMK2G HSPG2 EGFR ACTB ACTG1 |
| Phagosome | 11 | 0.00536717 | TUBB2B LAMP1 ITGA2 TUBB SLA-2 LOC100624785 ITGAV CYBA CALR ACTB ACTG1 |
| Regulation of actin cytoskeleton | 11 | 0.04031857 | ITGB4 ITGA2 F2R MSN ITGA6 ITGAV BAIAP2 EGFR ACTB ACTG1 SSH1 |
| Steroid biosynthesis | 8 | 8.18E-07 | SQLE EBP SC5D MSMO1 DHCR24 LSS CYP51 FDFT1 |
| Glycolysis / Gluconeogenesis | 8 | 6.54E-04 | GPI LDHA PKM ACSS2 TPI1 PGK1 GAPDH HK1 |
| Fatty acid metabolism | 7 | 0.001245585 | ELOVL5 SCD FASN HADH HACD3 ACAT2 ACACA |
| Biosynthesis of amino acids | 7 | 0.005937287 | PKM TPI1 ACY1 IDH1 PGK1 GAPDH GLUL |
| Arrhythmogenic right ventricular cardiomyopathy (ARVC) | 7 | 0.006428675 | TCF7L1 ITGB4 ITGA2 ITGA6 ITGAV ACTB ACTG1 |
| Glucagon signaling pathway | 7 | 0.043891441 | LDHA PKM SLC2A1 CALM3 ITPR3 CAMK2G ACACA |
| Pyruvate metabolism | 6 | 0.003005758 | LDHA PKM ACSS2 ME1 ACAT2 ACACA |
| Mucin type O-Glycan biosynthesis | 5 | 0.006460422 | GALNT7 GALNT2 GALNT18 ST3GAL1 B4GALT5 |
| Glutathione metabolism | 5 | 0.048833489 | RRM2 GPX4 IDH1 MGST3 PGD |
| Terpenoid backbone biosynthesis | 4 | 0.015961228 | FDPS MVK HMGCS1 ACAT2 |
| Butanoate metabolism | 4 | 0.023368492 | HMGCS1 HADH ACAT2 AACS |
| Propanoate metabolism | 4 | 0.029176619 | LDHA ACSS2 ACAT2 ACACA |
